# 联合血清肿瘤标志物建立预测厄洛替尼治疗复治非小细胞肺癌生存模型

**DOI:** 10.3779/j.issn.1009-3419.2014.05.05

**Published:** 2014-05-20

**Authors:** 岚 邵, 卫 洪, 蕾 郑, 春晓 何, 贝贝 张, 发君 谢, 正波 宋, 广媛 娄, 沂平 张

**Affiliations:** 1 310022 杭州，浙江省肿瘤医院化疗中心 Department of Medical Oncology, Zhejiang Cancer Hospital, Hangzhou 310022, China; 2 310022 杭州，浙江省胸部肿瘤重点实验室 Key Laboratory Diagnosis and Treatment Technology on Thoracic Oncology, Hangzhou 310022, China; 3 310053 杭州，浙江中医药大学第二临床医学院 Zhejiang Chinese Medical University, the 2nd Clinical Medical College, Hangzhou 310053, China

**Keywords:** 肺肿瘤, 厄洛替尼, 血清肿瘤标志物, 预后因子, 预测模型, Lung neoplasms, Erlotinib, Serum tumor marker, Prognostic factor, Predictive model

## Abstract

**背景与目的:**

分子靶向治疗是肺癌个体化治疗的方向，目前已有学者建立靶向治疗预测模型，为临床个体化治疗提供更多的指导。本研究探讨血清肺表面活性物质相关蛋白（pulmonary surfactant-associated protein D, SP-D）、转化生长因子-α（transforming growthfactor α, TGF-α）、基质金属蛋白-9（matrix metalloproteinase 9, MMP-9）、组织多肽特异性抗原（tissue polypeptide specific antigen, TPS）、肺腺癌相关抗原（Krebs von den Lungen-6, KL-6）与晚期复治非小细胞肺癌（non-small cell lung cancer, NSCLC）治疗疗效及生存的关系，并构建生存预测模型。

**方法:**

采用酶联免疫吸附法（enzyme-linked immuno sorbent assay, ELISA）检测114例晚期复治NSCLC患者治疗前外周血清中SP-D、TGF-α、MMP-9、TPS、KL-6含量，结合临床因素分析与厄洛替尼治疗疗效的关系，采用*Kaplan-Meier*生存曲线、*Cox*多因素生存分析模型进行单因素和多因素分析，并构建生存预测模型。

**结果:**

114例患者厄洛替尼治疗总有效率为22.8%，稳定率为72.8%，中位无疾病进展时间（progression-free survival, PFS）为5.13个月，1年生存率为69.3%。SP-D > 110 ng/mL组的有效率及稳定率均高于≤110 ng/mL组（*P*=0.011, *P*=0.017），MMP-9≤535 ng/mL的稳定率高于 > 535 ng/mL组（*P*=0.009）。TPS < 80 U/L组的稳定率要高于≥80 U/L组（*P*=0.002）。SP-D > 110 ng/mL组的mPFS长于≤110 ng/mL组（5.95个月*vs* 3.25个月，*P*=0.009），MMP-9≤535 ng/mL的mPFS要长于 > 535 ng/mL组（5.83个月*vs* 3.47个月，*P*=0.046），KL-6中 < 500 U/mL组要优于≥500 U/mL组（6.03个月*vs* 3.40个月，*P*=0.040），TPS < 80 U/L组的mPFS要长于≥80 U/L组（6.15个月*vs* 2.42个月，*P*=0.014）。多因素分析显示吸烟史、*EGFR*基因野生型、末次化疗疗效进展、厄洛替尼治疗期间无皮疹、LDH升高和TPS≥80 U/L是PFS不佳的独立影响因素。通过建立预后预测模型，根据患者的预后指数可分成4组：低危组、中低危组、中危组和高危组，中位PFS分别是9.12个月、6.88个月、3.52个月和0.93个月，组间差异具有统计学意义（*P* < 0.001）。

**结论:**

血清肿瘤标志物TPS水平联合患者临床特征建立预测厄洛替尼治疗晚期复治NSCLC生存模型，在临床上有一定的指导意义。

## Abstract (en)

**Background and objective:**

Molecular targeting therapy is the direction of individualized treatment of lung cancer, scholars has been established targeted therapy prediction models which provide more guidance for clinical individual therapy. This study investigated the relationship among pulmonary surfactant-associated protein D (SP-D), transforming growth factor α (TGF-α), matrix metalloproteinase 9 (MMP-9), tissue polypeptide specific antigen (TPS), and Krebs von den Lungen-6 (KL-6) and response as well as survival in the patients with recurrent non-small cell lung cancer, which Erlotinib was as second line treatment after failure to chemotherapy. This study also established a predictive prognostic model.

**Methods:**

Serum levels of SP-D, TGF-α, MMP-9, TPS, and KL-6 in 114 patients before erlotinib treatment were detected by ELISA method. Combined with clinical factors, these levels were used to investigate the relationship with efficacy in erlotinib treatment and construct a predicted prognostic model by *Kaplan-Meier* curve and *Cox* proportional hazard model multivariate analysis.

**Results:**

The objective response rate (ORR) and disease control rate (DCR) in the 114 patients, were 22.8% (26/114) and 72.8% (83/114), to Erlotinib treatment respectively. The median progression-free survival (PFS) and one year survival rate with Erlotinib treatment were 5.13 months and 69.3%, respectively. Patients in the SP-D > 110 ng/mL group exhibited more ORR (33.3% *vs* 13.3%, *P*=0.011) and DCR (83.3% *vs* 63.3%, *P*=0.017) than those in the ≤110 ng/mL group. Patients in the MMP-9≤535 ng/mL group showed more DCR (83.9%) than those in the > 535 ng/mL group (62.1%) (*P*=0.009). Patients in the TPS < 80 U/L group showed more DCR (82.4%) than those in the ≥80 U/L group (55.0%) (*P*=0.002). The SP-D > 110 ng/mL (5.95 months *vs* 3.25 months, *P*=0.009), MMP-9≤535 ng/mL (5.83 months *vs* 3.47 months, *P*=0.046), KL-6 < 500 U/mL (6.03 months *vs* 3.40 months, *P*=0.040), and TPS < 80 U/L (6.15 months *vs* 2.42 months, *P*=0.014) groups showed better PFS. Multivariate analysis showed that current or ever-smoker, wild style of EGFR status, progression after prior chemotherapy, absence of skin rash, elevated serum LDH level, and TPS≥80 U/L were independent adverse prognostic factors for PFS. These six factors were used in the prognostic model. Patients were categorized into four prognosis risk groups based on the prognostic index from the model, namely, low risk, intermediate low risk, intermediate risk, and high risk groups. The median PFS of good, intermediate, poor, and very poor prognosis groups were 9.12, 6.88, 3.52, and 0.93 months (*P* < 0.001), respectively.

**Conclusion:**

The prognostic model based on clinical parameters with TPS will be useful in identifying patients who might be most likely to benefit from Erlotinib therapy in the patients with recurrent non-small cell lung cancer.

目前，癌症是全球疾病死亡的主要原因之一。据统计，2008年全世界因癌症死亡的有760万人，约占所有死亡人数的13%。其中，肺癌发生率最高，达到了137万例左右^[1]^。在我国，据《2012中国肿瘤登记年报》报道，2009年全国肿瘤的发病率为285.91/10万，肺癌占恶性肿瘤发病的第一位（约54/10万），死亡率也居首位（约46/10万）^[2]^。肺癌中大约80%是非小细胞肺癌（non-small cell lung cancer, NSCLC），预后较差，5年生存率仅为16%左右，且超过40%-50%的NSCLC患者在发现时已是晚期^[3]^，失去了手术的机会，化疗是其治疗的主要手段之一。传统的两药联合方案化疗缓解率为30%左右，中位生存时间仅8个月-11个月^[4]^。2004年，随着表皮生长因子受体酪氨酸激酶抑制剂（epidermal growth factor receptor tyrosine kinase inhibitor, EGFR-TKI）药物的问世，使*EGFR*基因突变肺癌患者的肿瘤缓解率提高到了60%左右，总体生存时间也达到了20多个月^[5, 6]^。厄洛替尼（特罗凯，Tarceva）是第一代可逆EGFR-TKI，BR.21^[7]^、TRUST^[8]^、TITAN^[9]^等研究逐步奠定了厄洛替尼在晚期NSCLC的二、三线治疗中的重要地位。

尽管靶向药物的治疗带来了更好的疗效和生存获益，但基因状态相同的患者也在疗效、有效时间和毒副反应方面存在差异。BR.21研究中发现与有效率高相关的指标有：女性、腺癌、亚裔人、不吸烟、EGFR蛋白表达阳性大于10%。多因素分析发现腺癌、亚裔人及不吸烟的患者生存期较长。已有学者利用临床因素和其他分子标志物筛选有意义的指标，并建立预后预测模型，为临床个体化治疗提供更多更便利的指导^[10-12]^。

目前临床上广泛开展的*EGFR*基因突变的检测方法，如直接测序法、焦磷酸测序法及ARMS法等都必须通过肿瘤组织检测，然而很多晚期肿瘤患者难以获得足量检测的肿瘤组织，从而限制了其个体化治疗的开展。临床上外周血的采集比较方便，尤其血清肿瘤标记物是目前研究的热门，我们希望能够通过有效的血清肿瘤标记物的检测来找到靶向药物疗效预测指标。目前已经有单中心研究观察了血清肺表面活性物质相关蛋白（pulmonary surfactant-associated protein D, SP-D）、转化生长因子-α（transforming growthfactor α, TGF-α）、基质金属蛋白-9（matrix metalloproteinase 9, MMP-9）、组织多肽特异性抗原（tissue polypeptide specific antigen, TPS）、肺腺癌相关抗原（Krebs von den Lungen-6, KL-6）等是其中一项标记的阳性预测价值，且有些是仅基于国外人群的研究，但未见这些标记观察的联合研究，更未见基于国内人群的联合观察研究。

基于此，我们检测了114例晚期复治NSCLC患者厄洛替尼治疗前外周血中以上五种血清肿瘤标志物水平，同步收集性别、吸烟史、体力状况评分、病理类型、治疗后有无皮疹、转移部位、既往化疗方案数目及疗效等临床资料，常规血液检测指标如血红蛋白（hemoglobin, Hb）、碱性磷酸酶（alkaline phosphatase, ALP）、乳酸脱氢酶（lactate dehydrogenase, LDH）、C反应蛋白（C-reactive protein, CRP）、细胞角蛋白19片段（Cytokerantin-19-fragment, CYFRA21-1）、癌胚抗原（carcinoembryonic antigen, CEA）水平等，分析这些因素与有效率（response rate, RR）、疾病控制率（rdisease control rate, DCR）及预后等的关系，并建立应用厄洛替尼治疗复治晚期NSCLC患者的疗效预测模型，为肺癌个体化治疗道路的探索提供依据。

## 1 资料与方法

### 1.1 临床资料

收集2008年7月-2012年9月在浙江省肿瘤医院化疗科接受厄洛替尼治疗的Ⅲb期或Ⅳ期复治NSCLC患者共114例。符合标准患者告知详情并签署知情同意书，入选患者在厄洛替尼治疗前1周内空腹采取静脉全血2 mL，置入促凝管，离心3, 000 rpm，10 min，收集血清，分装后置于-80℃超低温冰箱保存备测。

### 1.2 外周血清肿瘤标志物检测

SP-D、TGF-α、MMP-9、KL-6、TPS均采用采用酶联免疫吸附法（enzyme-linked immuno sorbent assay, ELISA）检测，利用Curve Expert 1.4软件绘制各血清肿瘤标志物的标准曲线图，再算出每个样本对应的浓度值。

### 1.3 治疗

所有患者均接受厄洛替尼（特罗凯，Tarceval，上海罗氏制药有限公司）单药治疗，口服，150 mg/d，允许减量2次，最低剂量50 mg/d。

### 1.4 近期疗效评价

所有患者接受厄洛替尼治疗4周复查评估近期疗效，对于疗效稳定或有效的患者，8周复查确定疗效，此后每2个月复查1次CT及进行其他影像学检查。疗效评价采用实体瘤疗效评价标准（Response Evaluation Ccriteria in Solid Tumors, RECIST）1.1^[13]^评价近期疗效，分为完全缓解（complete response, CR）、部分缓解（partial response, PR）、疾病稳定（stable disease, SD）和疾病进展（progressive disease, PD）。近期有效率RR＝（CR+PR）/（CR+PR+SD+PD）×100%。疾病控制率DCR＝（CR+PR+SD）/（CR+PR+SD+PD）×100%。

### 1.5 随访和生存分析

以首次用药起始时间为起点，疾病进展时间或任何原因死亡作为终点记录患者无疾病进展生存期（progression-free survival, PFS），对于在随访截止日期无进展的病例，在统计时作为删失数据处理。随访采用门诊随访或电话方式，末次随访时间为2013年2月。

### 1.6 不良反应评定

不良反应按照NCI-CTC 3.0 “抗癌药物常见毒性反应分级标准”，分为0度-4度。

### 1.7 统计学分析

统计软件采用SPSS PASW 18.0。MMP-9、KL-6、TPS、SP-D、TGF-α与年龄、性别、吸烟史、病理类型、肿瘤临床病理分期、化疗方案等因素的关联分析采用卡方检验。所有统计均采用双侧检验。PFS采用*Kaplan-Meier*法及*Cox*比例风险模型进行预后分析。*P* < 0.05为差异有统计学意义。

## 2 结果

### 2.1 临床资料特点

114例入选患者的一般情况：中位年龄55岁（25岁-82岁）， < 65岁患者比例较多（79.8%），91例，≥65岁23例；男性77例，女性37例；不吸烟与吸烟的患者比例接近（50.9% *vs* 49.1%）；体能状态（performance status, PS）评分0分-1分有83人，占72.8%，PS评分2分-3分占27.2%；病理类型主要分成两大类：腺癌69例，非腺癌45例；31.6%的患者明确了*EGFR*基因状态，其中突变23例，野生型13例；疾病分期以Ⅳ期为主，104例（91.2%），Ⅲb期10例（8.8%）；末次化疗疗效为CR/PR/SD的患者为99例（86.8），PD患者15例（13.2%）；既往只接受过一种化疗方案的患者64例（56.1%），≥2种化疗方案50例（43.9%）。详见表 1。

**1:** 114例Ⅲb/Ⅳ期复治非小细胞肺癌（non-small cell lung cancer, NSCLC）患者的临床特征 The clinical characteristics of 114 patients with stage Ⅲb/Ⅳ lung cancer failed to chemotherapy chemotherapy

Variable	*n* (114)	propotion (%)
Gender		
Female	37	32.5
Male	77	67.5
Median age at diagnosis		
< 65 year	91	79.8
≥65 year	23	20.2
Smoking characteristics		
Never smoker	58	50.9
Current or former smoker	56	49.1
Performance status		
0-1	83	72.8
2-3	31	27.2
Histology		
Adenocarcinoma	69	60.5
Non-adenocarcinoma	45	39.5
*EGFR* status		
Mutation	23	20.2
Wild	13	11.4
Unknown	78	68.4
Staging before erlotinib treatment		
Ⅲb	10	8.8
Ⅳ	104	91.2
Prior chemotherapy effect		
CR/PR/SD	99	86.8
PD	15	13.2
Prior chemotherapy		
One regimen	64	56.1
Two or more regimens	50	43.9
EGFR: epidermal growth factor receptor; CR: complete response; PR: partial response; SD: stable disease; PD: progressive disease.		

### 2.2 疗效反应

在114例接受厄洛替尼治疗的患者中，有1例患者获得CR，25例患者达到PR，总体RR为22.8%（26/114），DCR为72.8%（83/114）。

将各个临床因素进行分层，在RR方面：PS评分0分-1分的患者RR要高于PS评分2分-3分的患者（27.7% *vs* 9.7%, *P*=0.041）；病理类型是腺癌的患者有效率要高于非腺癌的患者（30.4% *vs* 11.1%, *P*=0.016）；*EGFR*基因突变的患者RR明显高于野生型和基因状态未知的患者（47.6% *vs* 0 *vs* 20.5%, *P*=0.002）；末次化疗疗效获得疾病控制的（即疗效为CR/PR/SD）的患者RR明显高于末次化疗疗效PD的患者（26.3% *vs* 0, *P*=0.024）；在厄洛替尼治疗期间出现皮疹的患者RR高于无皮疹的患者（30.1% *vs* 9.8%, *P*=0.013）。

在DCR方面：其中既往不吸烟的患者DCR要高于有吸烟史的患者（71.0% *vs* 64.3%, *P*=0.045）；病理类型是腺癌的患者DCR高于非腺癌的患者（81.2% *vs* 60.0%, *P*=0.013）；*EGFR*基因突变的患者DCR明显高于野生型、基因状态未知的患者（90.5% *vs* 33.3% *vs* 75.6%, *P* < 0.001）；末次化疗疗效获得疾病控制的（即疗效为CR/PR/SD）的患者DCR明显高于末次化疗疗效PD的患者（79.8% *vs* 26.7%, *P* < 0.001）；在厄洛替尼治疗期间出现皮疹的患者DCR高于无皮疹的患者（80.8% *vs* 58.5%, *P*=0.010）。

### 2.3 实验室常规检测指标与疗效的关系

同步收集114例患者厄洛替尼治疗前1周内实验室常规检测指标：Hb、ALP、LDH、CRP、CYFRA21-1、CEA，根据正常值范围分别分成正常组和异常组。分析其与厄洛替尼治疗疗效的关系，Hb正常组RR和DCR均高于异常组（32.9% *vs* 4.9%, *P* < 0.001）（82.2% *vs* 56.1%, *P*=0.003），差异有统计学意义。LDH正常组DCR高于异常组（79.0% *vs* 57.6%, *P*=0.020）。

### 2.4 血清肿瘤标志物与疗效的关系

114例患者厄洛替尼治疗前血清肿瘤标志物水平检测结果详见表 2。为了便于进一步与疗效及预后的分析，将患者每个血清肿瘤标志物按水平情况分成两组。通过查阅相关文献，SP-D的截点定为110 ng/mL^[14]^，TGF-α截点为12 pg/mL^[15]^，TPS截点为80 U/L^[16]^，MMP-9和KL-6分别以中位值定为截点（535 ng/mL, 332 U/mL）。

**2:** 114例Ⅲb/Ⅳ期肺癌患者血清肿瘤标志物水平 The serum levels of tumor biomarkers in the patients with stage Ⅲb/Ⅳ lung cancer

Item	Median	Range
SP-D (ng/mL)	59.35	9.74-357.39
TGF-*α* (pg/mL)	11.98	5.40-21.33
MMP-9 (ng/mL)	535.19	378.30-740.91
KL-6 (U/mL)	332.12	0-2, 949.21
TPS (U/L)	373.20	25.44-693.05
SP-D: pulmonary surfactant-associated protein D; TGF-*α*: transforming growthfactor *α*; MMP-9, matrix metalloproteinase 9; KL-6: Krebs von den Lungen-6; TPS: tissue polypeptide specific antigen.		

分析5项血清肿瘤标志物与患者厄洛替尼治疗疗效的关系发现，SP-D > 110 ng/mL组的RR及DCR均高于≤110 ng/mL组，RR分别是33.3%和13.3%（*P*=0.011），DCR分别是83.3%和63.3%（*P*=0.017）。MMP-9≤535 ng/mL的DCR要明显高于 > 535 ng/mL组（83.9% *vs* 62.1%, *P*=0.009）。TPS的 < 80 U/L组的DCR要高于≥80 U/L组（*P*=0.002）。TGF-α和KL-6组间的RR和DCR均没有统计学差异。

### 2.5 预后分析

114例晚期NSCLC复治患者接受厄洛替尼治疗的中位PFS是5.13个月（95%CI: 3.49-6.77），见图 1。1年生存率为69.3%。

**1:**
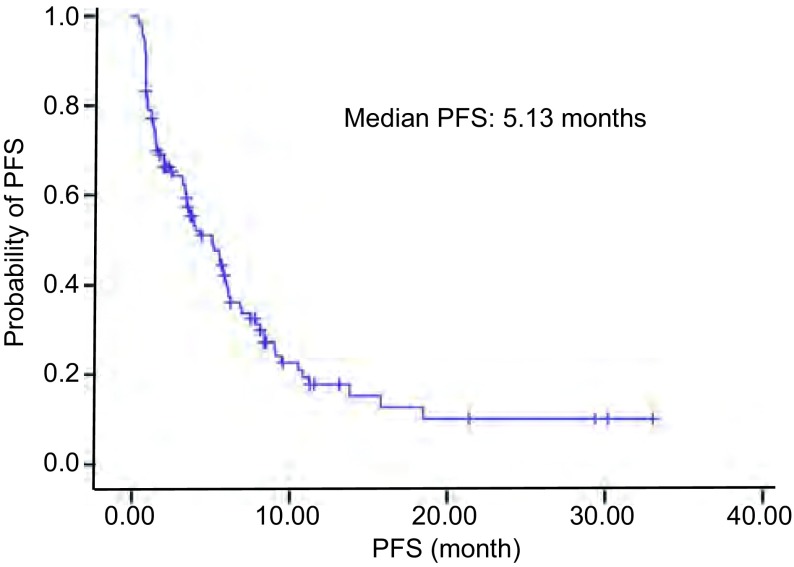
114例厄洛替尼治疗患者PFS曲线 The PFS curves of 114 Ⅲb/Ⅳ lung cancer patients received erlotinib treatment

通过*Kaplan-Meier*分析发现既往不吸烟的患者较吸烟患者mPFS明显延长（5.23个月*vs* 3.95个月，*P*=0.028）。病理类型为腺癌较非腺癌患者可获得较长的mPFS（5.95个月*vs* 3.25个月，*P*=0.002）。*EGFR*基因突变组mPFS可达15.8个月，明显长于野生组和未知组，野生组最短，仅1.03个月（*P* < 0.001）。末次化疗疗效获得疾病控制的患者较进展的患者mPFS长（5.82个月*vs* 1.03个月，*P* < 0.001）。在厄洛替尼治疗期间出现皮疹的患者较无皮疹的患者mPFS也要长（5.60个月*vs* 3.25个月，*P*=0.014）。在厄洛替尼治疗前1周内Hb检测值正常的患者较异常的患者mPFS长（5.83个月*vs* 1.72个月，*P*=0.001）。LDH正常的mPFS长（5.98个月*vs* 3.47个月，*P*=0.001）。CRP正常的也较异常的患者获得较长的mPFS（5.82个月*vs* 2.07个月，*P*=0.010）。

进一步分析检测的5项血清肿瘤标志物与患者厄洛替尼治疗mPFS的关系发现，SP-D > 110 ng/mL组的mPFS要长于≤110 ng/mL组（5.95个月*vs* 3.25个月，*P*=0.009），见图 2A。TGF-α两组间mPFS无明显统计学差异（5.6个月*vs* 4.07个月，*P*=0.579），见图 2B。MMP-9≤535 ng/mL的mPFS要长于 > 535 ng/mL组（5.83个月*vs* 3.47个月，*P*=0.046），见图 2C。KL-6中 < 500 U/mL组要优于≥500 U/mL组（6.03个月*vs* 3.40个月，*P*=0.040），见图 2D。TPS的 < 80 U/L组mPFS要明显长于≥80 U/L组（6.15个月*vs* 2.42个月，*P*=0.014），见图 2E。

**2:**
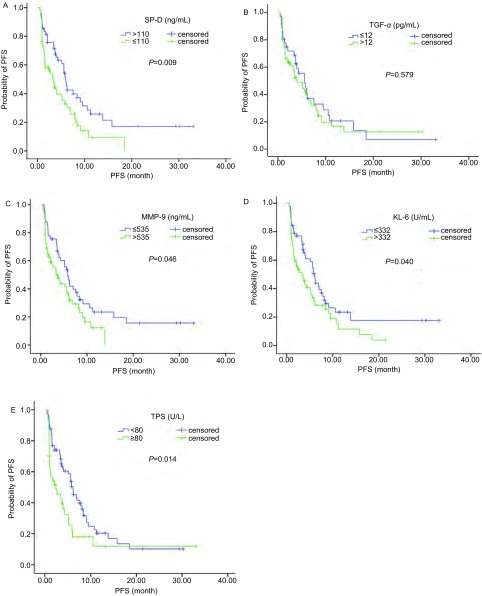
厄洛替尼治疗114例晚期复治NSCLC患者5种血清肿瘤标志物与PFS的关系。A：SP-D；B：TGF-*α*；C：MMP-9；D：KL-6；E：TPS。 The relationship among serum tumor markers and PFS in the 114 patients with recurrent non-small cell lung cancer treated with Erlotinib as second line therapy. A: SP-D; B: TGF-*α*; C: MMP-9; D: KL-6; E: TPS.

经*Cox*回归多因素分析5种血清肿瘤标志物与PFS的影响发现，SP-D（HR=1.865, 95%CI: 1.14-3.04, *P*=0.013）和TPS（HR=1.698, 95%CI: 1.03-2.79, *P*=0.037）是PFS的独立影响因素，见表 3。

**3:** 厄洛替尼治疗114例复发性非小细胞肺癌患者血清肿瘤标志物与PFS的多因素分析 Multivariate analysis of PFS and serum tumor markers in the 114 recurrent lung cancer patients treated with Erlotinib

	PFS	
	HR（95%CI）	*P*
SP-D	1.865 (1.14-3.04)	0.013
TGF-*α*	0.929 (0.56-1.55)	0.780
MMP-9	1.357 (0.85-2.18)	0.206
KL-6	1.253 (0.77-2.03)	0.359
TPS	1.698 (1.03-2.79)	0.037
PFS: progression-free survival.		

### 2.6 毒性反应

114例患者厄洛替尼治疗常见的不良反应是皮疹（64%）和腹泻（51.8%），1度较多，3度患者一般经过短期（不超过1周）的停药后症状可改善，有2例患者因反复3度皮疹后厄洛替尼减量至100 mg每天1次。有2例患者出现间质性肺炎，1例为1度，经暂停药物，抗生素和激素治疗后肺炎消失，继续厄洛替尼治疗后未再出现间质性肺炎。其中1例为2度间质性肺炎，并伴有气急、胸膜、咳嗽等症状加重情况，考虑患者年纪较大（> 75岁），经治疗肺炎消失后，家属停止了厄洛替尼治疗。其他常见不良反应如食欲下降、乏力、白细胞降低、肝功能损害的患者，经对症治疗后改善或恢复正常。详见表 4。

**4:** 厄洛替尼治疗114例晚期复治NSCLC肺癌患者的不良反应 The side effects of Erlotinib as second line treatment for 114 patient with recurrent non-small cell lung cancer

Toxicities	Toxic side effects				*n* (%)
	1	2	3	4	
Rash	36	29	8	0	73 (64.0)
Diarrhea	46	11	2	0	59 (51.8)
Anorexia	10	2	0	0	12 (10.5)
Fatigue	13	3	0	0	16 (14.0)
Hematologic	11	2	0	0	13 (11.4)
Hepatic	9	1	0	0	10 (8.8)
Interstitial lung disease	1	1	0	0	2 (1.8)

## 3 预测模型构建

选择单因素分析筛选出的影响PFS的12项变量作为观察指标，包括吸烟史、病理类型、*EGFR*基因突变状态、PS评分、末次化疗疗效、皮疹、Hb、LDH、SP-D、TPS、MMP-9、KL-6，进一步筛选出PFS的独立影响因素，将12项观察指标进行赋值，见表 5。应用*Cox*比例风险模型进行多因素分析，构建预测模型并验证，随后计算每个患者的个体预后指数（personal prognosis index, PI），并根据PI值进行预后分组，比较各组的PFS情况。以*P*≤0.05为差异有统计学意义。

**5:** 114例患者12项观察指标赋值表 12 indexes of 114 patients with recurrent non-small cell lung cancer

Index	Variable	Assignment
X1	Smoking characteristics	0: Never smoker; 1: Current or former smoker
X2	Performance status	0: 0-1; 1: 2-3
X3	Histology	1: Adenocarcinoma; 2: Non-adenocarcinoma
X4	*EGFR* status	1: Mutation; 2: Unknown; 3: Wild
X5	Prior chemotherapy effect	1: CR/PR/SD; 2: PD
X6	Rash	1: Yes; 2: No
X7	Hb	1: Normal; 2: Abnormal
X8	LDH	1: Normal; 2: Abnormal
X9	SP-D	1: > 110; 2: ≤110
X10	MMP-9	1: ≤535; 2: > 535
X11	KL-6	1: ≤332; 2: > 332
X12	TPS	1: < 80; 2: ≥80
Hb: hemoglobin; LDH: lactate dehydrogenase; MMP-9: matrix metalloproteinase 9; KL-6: Krebs von den Lungen-6; TPS: tissue polypeptide specific antigen.		

将12项观察指标应用*Cox*比例风险模型进行多因素分析，最后筛选出6项影响PFS有统计学意义的指标：吸烟史、*EGFR*基因状态、末次化疗疗效、皮疹、LDH、TPS。见表 6。

**6:** *Cox*多因素分析结果（逐步回归模型，Enter *P*≤0.05，Removal *P* > 0.10） Multivariate analysis of PFS (step by step regression model, Enter *P*≤0.05, Removal *P* > 0.10)

Enter variable	Regression coefficient	Standard error of regression coefficient	Wald test	*P*	Relative risk（RR）	RR (95%CI)	
						Minimum	Maximum
Smoking characteristics (X1)	0.622	0.239	6.780	0.009	1.863	1.166	2.975
EGFR status (X4)	0.561	0.161	12.087	0.001	1.753	1.277	2.404
Prior chemotherapy effect (X5)	1.441	0.344	18.571	< 0.001	4.226	2.194	8.140
Rash (X6)	0.793	0.238	11.072	0.001	2.209	1.385	3.523
LDH (X8)	0.933	0.258	13.104	< 0.001	2.543	1.543	4.215
TPS (X12)	0.778	0.245	10.080	0.001	2.176	1.347	3.517
RR: response rate.							

根据表 6的结果，利用*Cox*比例风险回归模型方法构建厄洛替尼治疗PFS预测模型：H (t, X) = h0 (t) exp (0.622X1 + 0.561X4 + 1.441X5 + 0.793X6 + 0.933X8 + 0.778X12)。

预后指数：

PI＝β0 + 0.622X1 + 0.561X4 + 1.441X5 + 0.793X6 + 0.933X8 + 0.778X12。

β0 = -(0.622X(——)1 + 0.561X(——)4 + 1.441X(——)5 + 0.793X(——)6 + 0.933X(——)8 + 0.778X(——)12)

= - 6.671。

将114例患者的PI值排序后，分别以PI值的25%、50%及75%位数作为截点，划分为低危组（≤-0.7538）、中低危组（> -0.7538且≤-0.2500）、中危组（> -0.2500且≤0.6150）及高危组（> 0.6150）。将114例患者分成4组后，进一步验证预测模型（表 7，图 3）。

**7:** 114例患者各风险组PFS情况 PFS of 4 risk groups according to the prognostic model

	mPFS	95%CI
Low risk group	9.12	7.001-11.239
Intermediate low risk group	6.88	5.581-8.179
Intermediate risk group	3.52	1.714-5.326
High risk group	0.93	0.896-0.971

**3:**
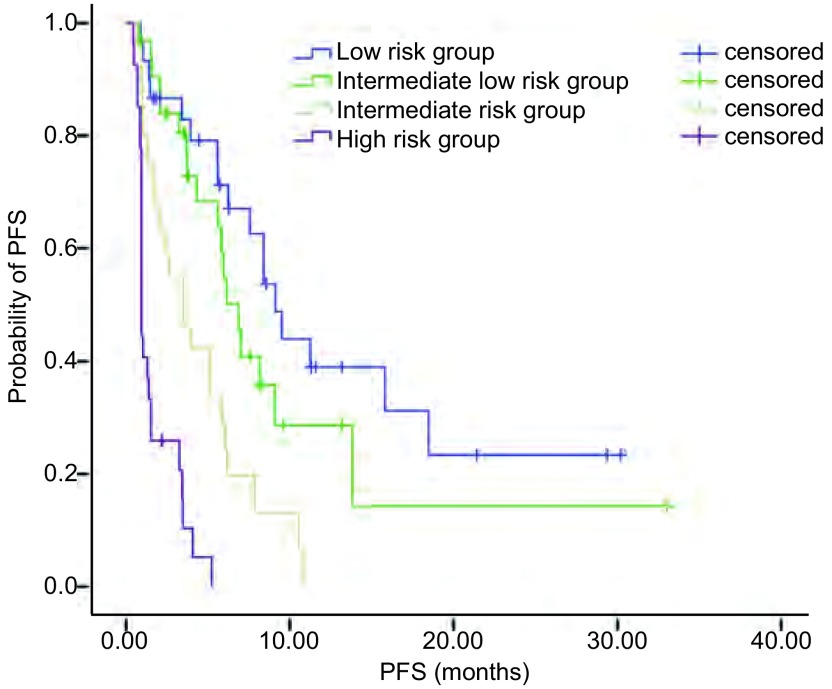
4组血清肿瘤标志物预测模型的PFS比较 Comparison of PFS among the 4 predictive models of serum tumor markers.

## 4 讨论

近年来，随着基因组、蛋白组学的发展和大量靶向药物的研究和应用，肺癌的治疗已经从传统的标准化治疗模式逐渐走上了以基因、蛋白为导向的个体化治疗的道路。第一代EGFR-TKI药物的出现不但为部分肺癌患者，特别是*EGFR*基因突变型患者带来了明显的生存获益和生活质量的改善，也加速了肺癌个体化治疗不断细化、不断完善的步伐。寻找新的靶点、新的肿瘤标志物也成为了当今肺癌治疗的热点，多个标志物或靶点的联合检测、联合治疗也是目前研究的方向之一。

厄洛替尼属于喹唑啉家族复合物，为可逆的ATP竞争性抑制剂。目前在临床上广泛的应用于*EGFR*基因19、21外显子突变的肺癌患者，其最常见不良反应为皮疹和腹泻，最严重的毒副作用为间质性肺炎。本研究共纳入114例Ⅲb期或Ⅳ期复治NSCLC患者接受厄洛替尼治疗，同时检测其治疗前外周血中TPS、TGF-α、SP-D、MMP-9和KL-6的水平，分析这5项血清肿瘤标志物、临床因素等与厄洛替尼治疗疗效及预后的关系。我们将5项指标进行了联合多因素分析，发现SP-D（HR=1.865, 95%CI: 1.14-3.04, *P*=0.013）和TPS（HR=1.698, 95%CI: 1.03-2.79, *P*=0.037）是影响晚期复治NSCLC患者厄洛替尼治疗PFS的独立影响因子，提示SP-D > 110 ng/mL和TPS < 80 U/L的患者可以获得较长的PFS。

本研究中114例患者接受厄洛替尼治疗总的RR为22.8%，DCR为72.8%。中位PFS为5.13个月，中位OS尚未达到。患者PS评分0分-1分、腺癌、*EGFR*基因突变、末次化疗疗效获得疾病控制的（即疗效为CR/PR/SD）、Hb正常、在厄洛替尼治疗期间出现皮疹的患者有效率更高。既往不吸烟、腺癌、*EGFR*基因突变、末次化疗疗效疾病控制的和有皮疹的患者可以获得更高的稳定率。在PFS方面提示既往不吸烟（*P*=0.028）、腺癌（*P*=0.002）、*EGFR*基因突变（*P* < 0.001）、末次化疗疗效获得疾病控制的（*P* < 0.001）、有皮疹（*P*=0.014）、LDH正常（*P*=0.001）和CRP正常（*P*=0.010）的患者PFS较长。

虽然厄洛替尼比传统的细胞毒化疗药物毒副反应小，治疗方便，但并不适用所有的肺癌患者，*EGFR*基因突变人群是治疗有效的靶点优势人群，RR可达70%以上^[17, 18]^，但如果是野生型患者RR则不足20%^[19]^，还不如化疗疗效佳。但临床上很多病例往往因肿瘤组织量太少而使*EGFR*基因检测受到限制。因此，如何尽可能挑选治疗有效的人群，避免盲目用药，使肿瘤治疗更具个体化成为医生和患者共同关注的问题。尽管目前已经有少量基于临床特征的预测厄洛替尼治疗NSCLC疗效的模型报道，但方法和结论也并不一致。

Florescu等^[10]^以BR.21研究人群为基础分析，提出了预测厄洛替尼治疗疗效的10项指标与OS相关，即吸烟史、PS评分、体重下降是否大于5%、是否贫血、是否乳酸脱氢酶升高、既往化疗方案的疗效、距离确诊肺癌的时间长短、既往化疗方案数、*EGFR*基因拷贝数和人种。根据每项临床因素的指数计算患者的预后指数，按预后指数将患者分成低危组、中低危组、中高危组和高危组。然后按照不同风险组，组内比较厄洛替尼治疗和安慰剂治疗的生存时间，最后发现低危组和中低危组内厄洛替尼治疗较安慰剂OS明显延长（*P* < 0.001, *P*=0.03），中高危组和高危组内厄洛替尼治疗和安慰剂治疗OS没有明显差别。由此得出结论，处于低危组和中低危组的患者可以更好地从厄洛替尼治疗中获益。

国内学者^[20]^将Florescu的预测模型用在中国人群中进行了检验，回顾性分析了119例接受吉非替尼治疗的晚期复治NSCLC患者，危险因素选取了10项因素中的8项（回顾性分析*EGFR*基因拷贝数和LDH水平资料不详），分成同样的4组，但因高危组仅3人，因此将中高危和高危组合并为一组。OS结果显示低危组未达到，低中危组8.9个月，中高危/高危组4.5个月。低危组OS明显优于其余两组（*P*=0.000, 3, *P*=0.000, 1），但低中危组和中高危/高危组之间的差异不具统计学意义（*P*=0.148）。造成结果的差异可能与人种不同，*EGFR*基因状态未纳入分析等有关，*EGFR*基因突变在亚裔人群中发生率要明显高于高加索人群^[21]^，*EGFR*基因突变状态^[22-24]^和拷贝数^[25]^是影响靶向治疗疗效和预后的重要因素。

Park等^[11]^将272例吉非替尼治疗肺癌患者进行多因素分析，筛选出PS评分2分-3分、腹腔内转移、ALP升高、从诊断到开始吉非替尼治疗≤12个月、白蛋白降低、之前化疗PFS≤12周、WBC > 10×10^9^/L和吸烟史这8项OS不良的预后风险因素。根据风险因素的个数进行分组，0-1个为预后良好组，2个-3个为预后中等组，4个-5个为预后差组， > 6个是预后极差组，mOS分别为18.0个月、11.2个月、4.0个月、1.3个月（*P* < 0.001）。

韩国Kim等^[12]^研究的模型跟Park的模型类似，通过研究257例特罗凯化疗的晚期NSCLC患者，筛选出具备以下一项与总生存时间较短相关，即：PS评分2分或以上、LDH升高及治疗后无皮疹，根据风险因素的个数：0个、1个、2个及3个分别为预后好、预后中等、预后差和预后极差4个组，每组中位OS分别为：22.0个月、9.3个月、5.4个月、2.7个月（*P* < 0.001）。具备以下一项与PFS短相关，即：腹腔内转移、接受过2个或以上方案化疗、治疗后无皮疹，同样根据风险因子个数分为4个组：预后好、预后中等、预后差和预后极差，中位PFS分别为：6.5个月、3.0个月、1.2个月、0.9个月（*P* < 0.001）。这种预测模型临床上使用较为方便，但每种风险因素的影响因子并不相同，仅以风险个数的多少来评定预后还是会出现偏移的情况，风险评定的准确性有待商榷。

我们在研究时先用单因素分析筛选影响厄洛替尼治疗PFS的因素，包括吸烟史、病理类型、*EGFR*基因突变状态、PS评分、末次化疗疗效、皮疹、Hb、LDH、SP-D、TPS、MMP-9、KL-6，再用*Logistic*回归对敏感因素进行多因素分析，筛选出吸烟史、*EGFR*基因状态、末次化疗疗效、皮疹、LDH、TPS这六个PFS的独立影响因素。利用*Cox*比例风险回归模型方法构建厄洛替尼治疗PFS预测模型，将114例患者的PI值排序后，分别以PI值的25%、50%及75%位数作为截点，划分为低危组、中低危组、中危组及高危组，mPFS分别为9.12个月、6.88个月、3.52个月、0.93个月（*P* < 0.001）。我们建模时利用*Cox*模型法可以细化每项因素的权重值，在分析结果时可以做到更个体化，也更准确。缺点就是在临床实践中相对不够直观，不太便利，在今后的下一步研究中我们会进一步完善，扩大样本量，在准确和便利中寻找合适的平衡点。另外，还需在更大的人群中进一步验证模型的准确性。
